# A risk-stratified model for predicting endometrial atypical hyperplasia and cancer to guide biopsy decisions in asymptomatic postmenopausal women

**DOI:** 10.3389/fmed.2025.1707883

**Published:** 2025-12-05

**Authors:** Shanshan Wu, Xu Zhang, Suhan Lai, Xiaohui Yang, Jing Wan

**Affiliations:** Department of Obstetrics and Gynecology, The Third Affiliated Hospital of Sun Yat-sen University, Guangzhou, China

**Keywords:** endometrial cancer, endometrial atypical hyperplasia, postmenopausal women, risk prediction model, endometrial thickness

## Abstract

**Background:**

Endometrial atypical hyperplasia (EAH) and endometrial cancer (EC) are increasingly detected in asymptomatic postmenopausal women. This often leads to delayed treatment. Risk stratification remains challenging, and single-factor models may not accurately identify high-risk individuals. This study aimed to develop and validate a multivariable prediction model for identifying EAH or EC in asymptomatic postmenopausal women.

**Methods:**

This retrospective cohort study included asymptomatic postmenopausal women with endometrial pathology records from the Third Affiliated Hospital of Sun Yat-sen University, China (2021–2024). Candidate risk factors included demographics, clinical characteristics, and hematological parameters. The primary outcome was a composite of histologically-confirmed EAH or EC. Multivariable Poisson regression with robust variance was then employed to identify independent risk factors for this composite outcome. Risk - stratified models were developed by calculating predicted probabilities for key combinations of risk factors.

**Results:**

Among 928 patients [median age: 59 years, IQR (interquartile range): 55–65; median BMI: 23.4 kg/m^2^], the overall prevalence of EAH and EC was 2.59% (24/928). Key independent risk factors included endometrial thickness (EMT) > 8 mm [vs. ≤ 4 mm: adjusted Relative Risks (aRR) = 11.34, 95% confidence interval (95% CI): 4.35–39.56; *p* < 0.001], diabetes (aRR = 2.54, 95% CI: 1.12–5.78; *p* = 0.026), and platelet count per 10^9^/L increase (aRR = 1.01, 95% CI: 1.01–1.02; *p* < 0.001). EMT > 8 mm was the strongest predictor, with the highest aRR. In the stratified analysis, the combination of EMT > 8 mm and diabetes was associated with the highest observed prevalence (33.33%). The risk-stratified model demonstrated clinical utility: using a ≥ 5% risk threshold, biopsy would be recommended for 17% of patients (sensitivity 70.8%, specificity 84.4%); at a ≥ 10% threshold, biopsy would be recommended for 4.3% of patients (requiring 3.64 biopsies per true positive case).

**Discussion:**

The risk of EAH and EC among asymptomatic postmenopausal women varies significantly based on clinical factors. This risk-stratified modeling approach delivers individualized risk estimates to inform endometrial biopsy decisions, facilitating personalized patient management.

## 1 Introduction

Endometrial cancer (EC) is one of the most common gynecological cancer, predominantly occurs in postmenopausal women. Driven by population aging and rising obesity rates, the global incidence of EC continues to rise. with 48,931 incident cases documented in China during 2024 (1, 2), while the number of new cases worldwide exceeds 420,000 annually (3). Although 90% of EC patients present with postmenopausal bleeding (4), approximately 5%–15% are diagnosed in asymptomatic women (5). In this subgroup, the absence of alarm symptoms frequently leads to delayed diagnosis, which is strongly associated with adverse prognostic outcomes.

The American College of Obstetricians and Gynecologists (ACOG) (6) and the British Gynaecological Cancer Society (BGCS) guidelines (7) recommend an EMT threshold of >4 mm for evaluating postmenopausal bleeding (PMB). Invasive endometrial sampling procedures should be employed to assess the risk of EC including aspiration (includes pipelle and endometrial biopsy), dilatation and curettage (D&C), and hysteroscopy. These procedures may induce patient anxiety (8), cause moderate-to-severe pain(8, 9), and entail risks of complications including endometritis, pelvic inflammatory disease (PID), and uterine perforation (9–11). However, the threshold of EMT for asymptomatic postmenopausal women remains controversial. Direct applicability of >4 mm EMT cut-off may lead to substantial overuse of invasive biopsy due to their lower baseline risk of malignancy (12). Some have suggested that an EMT of >8 mm measured by ultrasound (13) in asymptomatic populations may be a more optimal threshold (14, 15), but no consensus has been reached.

In addition to EMT, various clinically validated risk factors for EAH or EC include obesity, diabetes, metabolic syndrome, unopposed estrogen exposure (e.g., hormone replacement therapy), and parity. At the same time, blood markers (such as platelet count) (16) also show their prognostic potential.

However, relying solely on a single factor (e.g., EMT threshold) for risk assessment has obvious limitations and may lead to over-biopsy or missed diagnoses. Multifactorial risk prediction models may mitigate these limitations and demonstrate clinical utility in early cancer detection. There have been previous studies to develop EC multivariable prediction models for use in pre- and postmenopausal women. Both showed moderate to good discriminatory ability (C statistic 0.64 and 0.70) (17, 18), However, their development primarily using Western cohorts and incorporation of complex or costly biomarkers (e.g., genetic markers). Furthermore, they are used for primary EC prevention and failed to include EMT as the most important variable.

Therefore, this study aims to: (1) identify independent risk factors and develop a multivariable prediction model for EAH or EC in Chinese postmenopausal women, and then (2) generate individualized risk stratification estimates.

## 2 Materials and methods

### 2.1 Population selection

This cross-sectional retrospective study included naturally postmenopausal women who underwent endometrial sampling at the Gynecology Department of The Third Affiliated Hospital of Sun Yat-sen University between 1 January 2021, and 31 December 2024.

Medical records of all eligible patients were systematically reviewed. Participants meeting all inclusion criteria and none of the exclusion criteria were enrolled. Inclusion criteria were: (1) age ≥ 45 years with natural menopause [defined as ≥ 12 months of amenorrhea, excluding cases of bilateral oophorectomy or medications (e.g., GnRH analogs)]; (2) complete endometrial sampling pathology report available; (3) asymptomatic status (absence of vaginal bleeding, vaginal discharge, and pelvic pain). Exclusion criteria were: (1) duplicate encounters or individuals (retaining only the encounter with the most severe pathology); (2) missing EMT data; (3) history of hematological or immunological diseases; (4) current or recent tamoxifen use.

**FIGURE 1:**
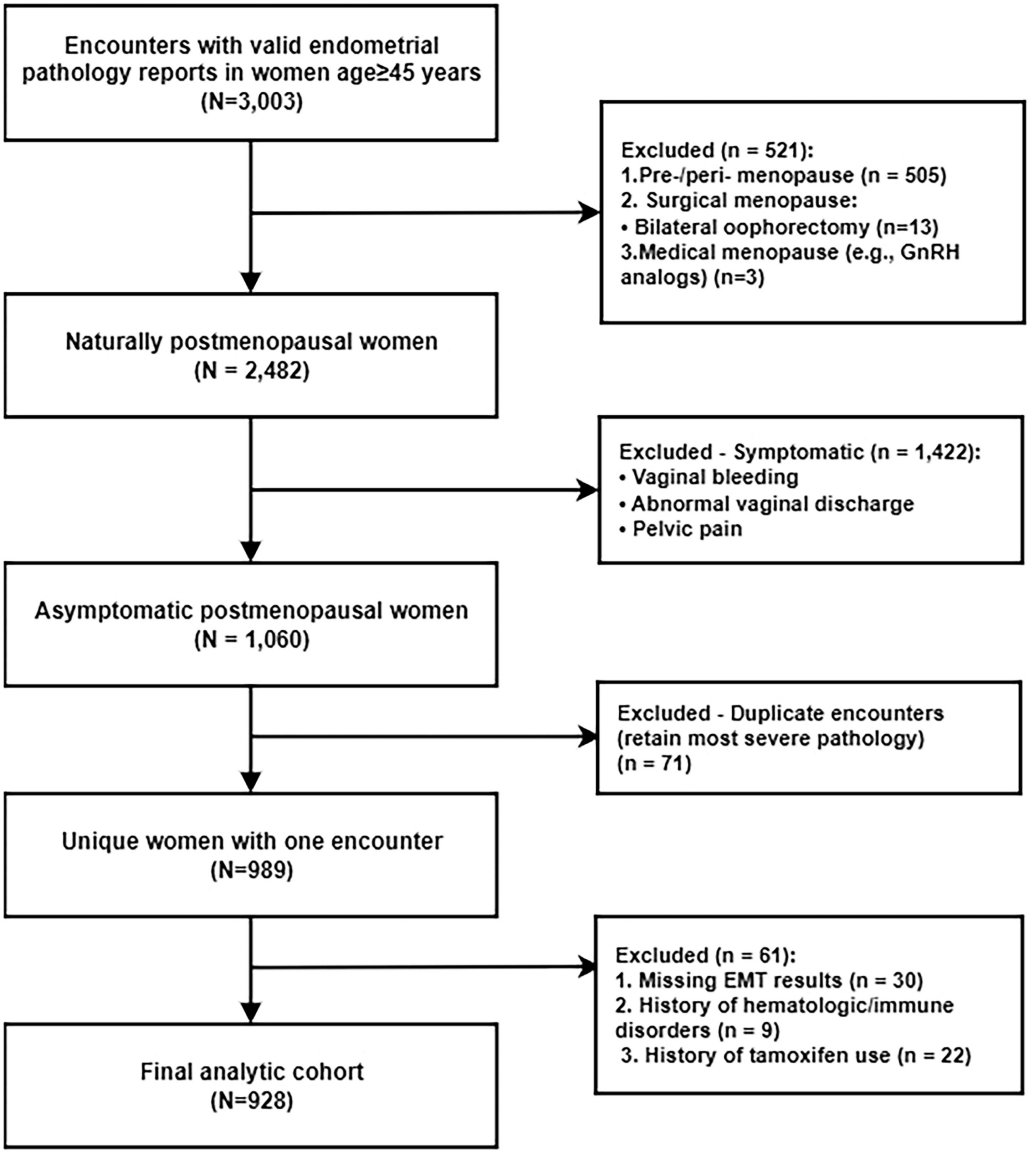
Flowchart of participant selection. EMT, endometrial thickness. Diagram illustrates the process of identifying the final study cohort of asymptomatic postmenopausal women from the initial patient population.

Among 3,003 patients aged ≥ 45 years who underwent endometrial sampling during the study period, we first identified naturally postmenopausal individuals. After excluding 1,422 women reporting vaginal bleeding, abnormal discharge, or pelvic pain (symptomatic), 1,060 asymptomatic subjects remained. We then excluded 71 duplicate records, 30 women with missing EMT data, nine with hematologic or immune disorders, and 22 who had used tamoxifen, leaving 928 unique asymptomatic postmenopausal women for final analysis (Figure 1).

All the procedures performed in studies involving human participants were in accordance with the ethical standards of Ethics Committee of IPMCH and with the 1964 Helsinki declaration and its later amendments or comparable ethical standards.

### 2.2 Definition of outcome

Our primary outcome comprised histologically-confirmed endometrial EAH or EC. We have reviewed the complete pathology reports, and categorized the findings into the specific pathological types listed, which includes Normal endometrium/Atrophic endometrium, Endometrial polyp, Simple/complex hyperplasia without atypia, Endometrial hyperplasia with polyp, EAH and EC. The distribution and classification of pathological results are shown in Table 1.

**TABLE 1:** Pathological classification of endometrial sampling results.

Pathology result	Total	*N* (%)
Normal endometrium/Atrophic endometrium	674	(72.6)
Endometrial polyp	217	(23.4)
Simple/complex hyperplasia without atypia	10	(1.1)
Endometrial hyperplasia with polyp	3	(0.3)
Atypical hyperplasia	5	(0.5)
Endometrial cancer	19	(2.1)

### 2.3 Statistical analyses

Descriptive statistics were used to report frequencies and percentages of baseline characteristics. Pathology Prevalence refers to the proportion of patients within each characteristic category who had EAH or EC.

To address the rare outcome, we used Poisson regression with robust variance to analyze risk factor, which is suitable for modeling rare events and adjusting for overdispersion, and less biased than logistic regression for common outcomes.

The following candidate variables were considered: Demographic characteristics [age (years), ethnicity (han ethnicity/other ethnic minorities), occupation (employed/unemployed/retired), education level (illiterate or primary school/secondary school/college or above)]; Clinical characteristics [BMI, maternal history (gravidity/parity), menopausal parameters (age/years), metabolic disorders (diabetes/hypertension), hormone replacement therapy (HRT), history of breast cancer]; Sonographic measure (EMT); and Hematological indicators (complete blood count parameters, coagulation profile, neutrophil-to-lymphocyte ratio (NLR), platelet-to-lymphocyte ratio (PLR), lymphocyte-to-monocyte ratio (LMR)). Univariate analyses were initially conducted to estimate crude relative risks (RR) with 95% confidence intervals (CI). Variables demonstrating a univariate association with *P* < 0.05 were subsequently entered into a multivariable Poisson regression model to derive adjusted relative risks (aRR) and 95% CIs. Multicollinearity was assessed using variance inflation factors (VIF), with a VIF < 5 indicating no substantial collinearity (Supplementary Table 4).

Sensitivity analyses included: (1) Testing interaction effects between EMT and diabetes mellitus, age at menopause, and platelet count; (2) Restricting outcomes to EC (excluding EAH) to test robustness against premalignant lesion heterogeneity; and (3) Analyzing EMT’s effects in different models: first adjusting only for demographic covariates (age, ethnicity, occupation, education), then metabolic and hormone exposure factors (BMI, diabetes, hypertension, age at menopause, duration of menopause), and finally all covariates from the multivariable analysis.

In the risk stratification analysis, continuous variables were categorized for stratification: platelet count (cut-off: 300 × 10^9^/L, based on prior studies demonstrating its prognostic relevance in solid tumors (19–21)), and age at menopause (cut-off: 50 years, aligned with the median menopausal age in the study population). For each combination of these stratified factors alongside EMT categories (≤4, >4 to ≤8, >8 mm), the following were calculated: (i) Prevalence of EAH or EC and corresponding 95% CI using the Clopper-Pearson exact method; (ii) Predicted prevalence based on the multivariable Poisson regression model; and (iii) Proportion of patients exceeding ≥5% or ≥10% risk thresholds to evaluate screening strategy efficacy.

Given the retrospective design and use of de-identified data, the study protocol was approved by the Ethics Review Committee of the Third Affiliated Hospital of Sun Yat-sen University, exempting informed consent.

All analyses were performed using SPSS 25.0 and R 4.4.2 (statistical significance: two-tailed *P* < 0.05).

## 3 Research results

### 3.1 Characteristics of the study population

This study ultimately enrolled 928 asymptomatic postmenopausal women. Baseline characteristics were as follows: median age 59 years [interquartile range (IQR): 55–65], median age at menopause 50 years (IQR: 49–52), median duration of menopause 8 years (IQR: 4–15), and mean body mass index (BMI) 23.4 kg/m^2^ (Table 2). Among patients with diabetes, the prevalence of EAH or EC was 6.67%, while among those with hypertension, it was 2.52%. Median EMT is 4.0 mm (IQR: 3.00–6.96 mm). Among the 16.49% (*n* = 153) of patients with EMT > 8 mm, the prevalence of EAH or EC was 10.46%. Within the overall cohort (*n* = 928), 24 cases of EAH or EC were identified, yielding an overall prevalence of 2.59%. This comprised 19 cases of EC (2.05%) and 5 cases of EAH (0.54%).

**TABLE 2:** Baseline characteristics of the study population.

Characteristic	*N*	%
Age (years) (IQR)	59.0	(55.0, 65.0)
**Ethnicity**		
Han	917	(99)
Other minorities	11	(1)
**Occupation**		
Employed	247	(27)
Unemployed	252	(27)
Retired	429	(46)
**Education level**		
Illiterate or primary school	331	(36)
Secondary school	384	(51)
College or above	213	(23)
BMI (IQR)	23.4	(21.3, 25.3)
Gravidity (IQR)	3.0	(2.0, 4.0)
Parity (IQR)	2.0	(1.0, 3.0)
Age at menopause (years) (IQR)	50.0	(49.0, 52.0)
Time since menopause (years)	8.0	(4.0, 15.0)
**Diabetes**		
No	823	(89)
Yes	105	(11)
**Hypertension**		
No	603	(65)
Yes	318	(35)
**HRT**		
No	865	(93)
Yes	8	(1)
**History of breast cancer**		
No	838	(90)
Yes	60	(7)
EMT (IQR)	4.0	(3.0,6.5)
**EMT categories**		
≤4 mm	585	(63)
>4 to ≤8 mm	190	(20)
>8 mm	153	(17)

IQR, interquartile range; EMT, endometrial thickness; BMI, body mass index; EAH, endometrial atypical hyperplasia; EC, endometrial cancer; HRT, hormone replacement therapy.

### 3.2 Risk factors for EAH or EC

Univariate Poisson regression analysis assessed demographic, clinical, EMT measured via ultrasound, and hematological indicators for their association with EAH or EC risk. Significant predictors identified included age at menopause, diabetes, EMT > 8 mm, and platelet count (all *p* < 0.05). The results of the univariate analysis for all candidate variables are presented in Table 3. In the subsequent multivariable Poisson model incorporating these significant variables, it was further confirmed that EMT was the strongest predictive factor—patients with EMT > 8 mm had a 11.34-fold increased risk of EAH or EC compared to the EMT ≤ 4 mm group (*p* < 0.01). Diabetes increased the risk of EAH or EC (aRR = 2.54, 95% CI: 1.12–5.78). Blood markers had limited predictive value, with only platelet count showing a weak association (aRR = 1.01, 95% CI: 1.01–1.02). The complete results of the multivariable analysis are summarized in Table 3. Although age at menopause was significant in the univariate Poisson regression, it did not show significance in the multivariate analysis. Considering its clinically observed impact, we will still include age at menopause in the subsequent model. Other hematological and coagulation parameters analyzed (e.g., PT, APTT, WBC) showed no significant association with EAH or EC risk in univariate analysis (Supplementary Table 1).

**TABLE 3:** Univariate and multivariate Poisson regression analysis of endometrial atypical hyperplasia and cancer risk factors.

	Univariate analysis		Multivariate analysis	
	RR [95% CI]	*P*	RR [95% CI]	*P*
**EMT categories**				
≤4 mm	–	–	–	–
>4 to ≤8 mm	1.85 [0.45, 7.66]	0.398	1.84 [0.45, 7.57]	0.399
>8 mm	12.24 [4.55, 32.87]	<0.001	11.34 [4.35, 39.56]	0.001
Diabetes (ref: 0)	3.23 [1.34, 7.60]	0.007	2.54 [1.12, 5.78]	0.026
Age at menopause	1.14 [1.01, 1.29]	0.045	1.12 [0.99, 1.28]	0.072
Platelet count	1.01 [1.01, 1.02]	0.011	1.01 [1.01, 1.02]	0.008
Age	1.01 [0.96, 1.06]	0.825	–	–
Ethnicity (ref: 1)	0 [0, inf]	0.991	–	–
**Occupation**				
Employed	–	–	–	–
Unemployed	0.49 [0.12, 1.94]	0.309	–	–
Retired	1.44 [0.57, 3.66]	0.445	–	–
**Education level**				
Illiterate or Primary school	–	–	–	–
Secondary school	1.51 [0.64, 3.5]	0.347	–	–
College or above	0.39 [0.08, 1.81]	0.123	–	–
BMI	0.98 [0.88, 1.09]	0.785	–	–
Gravidity	0.97 [0.66, 1.44]	0.886	–	–
Parity	1.15 [0.79, 1.67]	0.457	–	–
Time since menopause	0.98 [0.93, 1.04]	0.579	–	–
Hypertension (ref: 0)	0.95 [0.41, 2.19]	0.901	–	–
HRT (ref: 0)	0 [0, inf]	0.988	–	–
History of breast cancer	0 [0, inf]	0.992	–	–
NLR	0.91 [0.61,1.36]	0.652	–	–
LMR	0.98 [0.94,1.02]	0.270	–	–
PLR	1 [0.99,1.01]	0.696	–	–

The final multivariable Poisson regression model included variables with a univariate association of *p* < 0.05. Platelet count is expressed per 10^9^/L increase. RR, relative risk; CI, confidence interval; p, *p*-value; EMT, endometrial thickness; BMI, body mass index; NLR, neutrophil-to-lymphocyte ratio; PLR, platelet-to-lymphocyte ratio; LMR, lymphocyte-to-monocyte ratio.

### 3.3 Sensitivity analyses

Sensitivity analyses confirmed the robustness of the primary findings. First, poisson regression revealed significant interactions between EMT and diabetes (RR = 1.07, 95% CI: 1.05–1.09), age of menopause (RR = 1.003, 95% CI: 1.001–1.004), and platelet count (PLT) (RR = 1.001, 95% CI: 1.001–1.002) (all *p*-values < 0.05). For the EMT × diabetes interaction, the RR of 1.07 indicates that for every 1-mm increase in EMT, the risk of EAH or EC is an additional 7% higher in diabetic women than in non-diabetic women, demonstrating a synergistic effect. Second, when the outcome was restricted solely to EC, the key predictive factors remained stable. Finally, cross models with stepwise covariate adjustment, EMT > 8 mm consistently demonstrated the strongest predictive power (all *p*-values < 0.02). Across stepwise-adjusted models, EMT > 8 mm consistently showed the highest predictive power (all *p*-values < 0.02). The metabolic-hormonal model yielded an aRR (13.07) closest to the primary model (Supplementary Table 2), supporting synergistic risk amplification by metabolic factors and hormonal exposure duration.

### 3.4 Observed prevalence of EAH or EC stratified by independent risk factors

Endometrial thickness emerged as the strongest independent predictor for diagnosing EAH or EC in this cohort. Subsequent stratification by diabetes status, elevated platelet count (≥300 × 10^9^/L), and menopausal age (≥50 years) revealed distinct risk patterns. In the group with EMT ≤ 4 mm, the prevalence of EAH or EC was below 1.1% (range 0%–1.08%). When EC increased to 4–8 mm, the prevalence of EAH or EC in patients with diabetes significantly rose to 5.26%, and the prevalence in all subgroups also increased compared to the EMT ≤ 4 mm group (except menopausal age ≥ 50 years). For the EMT > 8 mm group, the baseline prevalence of EMT was 10.46% (95% CI: 6.10%–16.43%); when combined with diabetes, the prevalence sharply increased to 33.33% (95% CI: 13.34%–59.01%); if combined with platelet count ≥ 300 × 10^9^/L, the prevalence reached 24% (95% CI: 9.36%–45.13%) (Table 4).

**TABLE 4:** Observed prevalence of endometrial atypical hyperplasia (EAH) or endometrial cancer (EC) stratified by endometrial thickness (EMT) and comorbidities.

	EMT categories		
Overall [*N* = 928]	≤4 mm	>4 to ≤8 mm	>8 mm
	0.85% [0.28%, 1.98%]	1.58% [0.33%, 4.54%]	10.46% [6.10%, 16.43%]
**Diabetes**			
No	0.97% [0.31%, 2.24%]	1.17% [0.14%, 4.16%]	7.41% [3.61%, 13.20%]
Yes	–	5.26% [0.13%, 26.03%]	33.33% [13.34%, 59.01%]
**Age at menopause (years)**			
<50	0.51% [0.01%, 2.81%]	2.82% [0.34%, 9.81%]	12.00% [4.53%, 24.31%]
≥50	1.05% [0.29%, 2.67%]	0.86% [0.02%, 4.71%]	9.80% [4.80%, 17.29%]
**Platelet count (10^9^/L)**			
<300	0.99% [0.27%, 2.50%]	1.52% [0.18%, 5.37%]	9.62% [4.71%, 16.97%]
≥300	1.08% [0.03%, 5.85%]	3.33% [0.08%, 17.22%]	24.00% [9.36%, 45.13%]

### 3.5 Predicted prevalence and risk threshold benefit analysis

**FIGURE 2:**
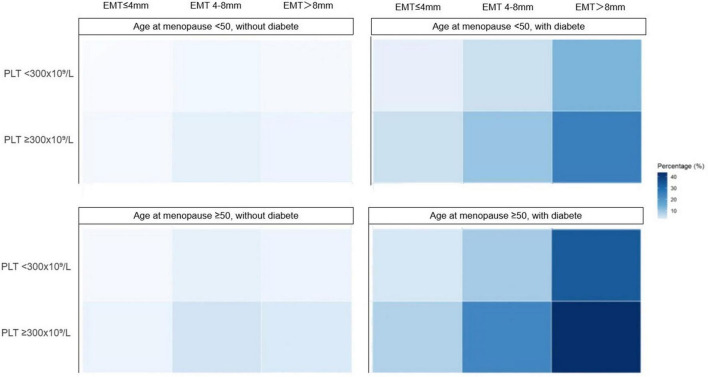
Predicted prevalence of endometrial atypical hyperplasia or cancer based on risk stratification models. EMT, endometrial thickness; PLT, platelet count. Predicted probabilities were derived from the multivariable Poisson regression model.

Using a robust multivariate Poisson regression model, we provide a visual representation of the predicted probabilities of EAH or EC for each combination of risk factors, including EMT, diabetes, PLT and age of menopause. These predictions are displayed visually in Figure 2. The highest-risk profile (EMT > 8 mm combined with diabetes, menopausal age ≥ 50 years, and platelet count ≥ 300 × 10^9^/L) yielded a predicted prevalence of 43.58%. Conversely, the lowest-risk profile (EMT ≤ 4 mm without diabetes, menopausal age < 50 years, and platelet count < 300 × 10^9^/L) demonstrated a predicted prevalence of 0.47%. The discriminative ability of the risk-stratified model, as measured by the area under the receiver operating characteristic curve (AUC), was 0.83 (95% CI: 0.73–0.92).

Considering a ≥ 5% risk threshold, 17% of patients would have met criteria to undergo endometrial sampling. All patients with EMT > 8 mm (risk range 5.32%–43.58%) met this threshold. Among the patients in the EMT 4–8 mm group, those with diabetes who also have either a platelet count ≥ 300 × 10^9^/L or an age at menopause ≥50 years are also included in this risk threshold model. This strategy achieved a sensitivity of 70.8% (17/24 true positive cases detected) and a specificity of 84.4% (763/904 true negative cases correctly spared biopsy). The positive predictive value (PPV) was 10.8% and the negative predictive value (NPV) was 99.1%. Compared to the strategy based solely on EMT > 4 mm, the biopsy rate was significantly reduced. Furthermore, compared to the strategy based solely on EMT > 8 mm, sensitivity increased by approximately 4%. (Supplementary Table 3)

Considering a ≥ 10% risk threshold, 4.3% of patients would have met criteria to undergo endometrial sampling. This strategy specifically targets the highest-risk subgroup, selecting only patients with EMT ≥ 8 mm who also meet at least one of the following criteria: (1) with diabetes (any age or platelet level, minimum risk 13.42%), or (2) platelet count ≥ 300 × 10^9^/L (any age or diabetes status, minimum risk 10.49%). Compared to the strategy based solely on EMT > 8 mm, the biopsy rate was further reduced by 12.2% (absolute reduction), and the number of biopsies required per true positive case detected was optimized to 3.64. However, this improvement in efficiency and reduction in biopsy burden came at the cost of a reduction in sensitivity (13 positive cases missed). For this strategy, the PPV was 27.5% and the NPV was 98.5%. (Supplementary Table 3).

## 4 Discussion

In this retrospective cohort of 928 asymptomatic postmenopausal women, we performed Univariate Poisson regression analysis to assessed the associations between EAH or EC risk and various factors, including demographic features, clinical variables, ultrasound-EMT, and hematological parameters. Among these, EMT > 8 mm, menopausal age, diabetes mellitus, and platelet count emerged as significant predictive factors.

Endometrial thickness is consistently reported as the strongest independent predictor of EAH or EC, as demonstrated in multiple studies (13, 22). Current ACOG and BJOG guidelines (5, 6) for women with post-menopausal bleeding regard an EMT < 4 mm as carrying a ≥ 99% negative predictive value for EC and therefore recommend biopsy only when EMT exceeds this limit. Whether the same 4 mm rule can be applied to asymptomatic women remains prompting a growing body of research aimed at defining the optimal EMT threshold in the absence of bleeding (13, 23). An Italian prospective cohort suggested that incidentally detected EMT > 8 mm warrants biopsy (11) and a large Chinese cross-sectional study identified 8 mm as the cut-off that maximized the AUC (0.715) (22). Some articles even advocate for a greater endometrial cutoff value for diagnosis (24, 25). To date, however, no consensus has been reached. In our study, EMT > 8 mm emerged as the strongest independent predictor of endometrial atypical hyperplasia or cancer (EAH or EC; aRR = 12.27).

Population-based studies show that women with diabetes have roughly a 70% higher risk of EC (26). Diets that protect against diabetes—low in refined carbohydrates, saturated fat and added sugar—also appear to reduce EC incidence.(27) The common denominator is insulin resistance, which amplifies estrogen signaling and creates a pro-tumor micro-environment.(28) Hyperinsulinaemia acts directly on endometrial epithelium via the insulin and IGF-I receptors to trigger mitogenic pathways (29) and indirectly by raising IGFs, altering sex-steroid availability and perturbing adipokine profiles that govern proliferation and apoptosis (30–33). Our finding of a marked synergistic increase in EC risk is therefore biologically plausible and consistent with the current literature.

A meta-analysis restricted to Asian women showed that every additional year after age 50 at menopause raises endometrial-cancer risk by ≈ 5% (34). The mechanism is thought to be prolonged, unopposed estrogen: persistent proliferative signaling expands the endometrial stem-cell pool, accumulating mutations that heighten malignant risk (35, 36). Anovulatory cycles common in the perimenopause aggravate the imbalance by depriving the endometrium of the countervailing action of progesterone (37, 38). Although age at menopause did not reach statistical significance in the multivariable model, we observed a trend toward increased risk with later menopause, which is consistent with the biological hypothesis that prolonged, unopposed estrogen exposure contributes to endometrial carcinogenesis.

Platelet counts contribute to tumor progression through the release of pro-angiogenic factors and have been widely utilized as a prognostic indicator in various tumors in recent years (39). Elevated platelet levels (300 × 10^9^/L) are associated with poor prognosis in colorectal cancer (40), thyroid cancer (41), and gynecological cancer (42–44). In EC, previous studies have further demonstrated that elevated platelet counts correlate with advanced disease, lymph node involvement, advanced FIGO stage, and poor disease-specific survival (45, 46). Our findings thus align with this evidence, confirming that platelet count serves as an independent predictive factor within our risk-stratified model.

The results of this study demonstrate no statistically significant association between BMI and EC incidence. This lack of association contrasts with the conclusions of most previous studies. First, while about 40% of EC cases were due to overweight and obesity in developed or industrialized countries (47), the mean baseline BMI in this study cohort was comparatively low (23.4 kg/m^2^), resulting in a significantly lower obesity prevalence and likely contributing to the null finding. Second, Asian populations exhibit a distinct pattern of central adiposity (48), suggesting that waist circumference or waist-to-hip ratio may represent more sensitive indicators of EAH or EC risk than BMI.

Although numerous studies have identified associations between various risk factors and EAH or EC, current diagnostic protocols for asymptomatic postmenopausal women remain limited to ultrasound-EMT as the sole criterion for biopsy referral (14, 24). Few EC risk prediction models which intergrated multi- epidemiological factors have previously been published. The E2C2 (17) and the Predicting risk of EC in asymptomatic women model (PRECISION) (18), showed good performance in quantifying a woman’s 10-year risk of EC. However, E2C2 model (17), which incorporates transcriptomic markers, was derived from a cohort of White women aged 45–85 years. Its reliance on costly genetic testing hinders widespread clinical adoption. The PRECISION model (18), was also developed in Western cohorts with a high prevalence of obesity, limiting its generalizability. Their use could determine eligibility for primary EC prevention. Neither model incorporates EMT, failing to provide direct guidance on which specific populations should be referred for biopsy. While some studies (49) in Asian populations have investigated postmenopausal biopsy strategies, their focus has largely remained on evaluating the predictive value of EMT as a single indicator. In our study, we developed the first stratified risk model for an Asian population to predict the risk of EAH or EC and guide biopsy decision making in asymptomatic postmenopausal women. Our model overcomes the limitation of single-indicator reliance by integrating EMT with other key clinical factors, demonstrated superior discriminative performance, with an AUC of 0.82.

We propose two distinct risk-stratified clinical strategies based on this model. Had the ≥5% risk threshold been applied (endometrial biopsy is recommended when the EC or EAH estimate risk ≥ 5%), only 158 patients would have required biopsy. It achieves higher specificity than the EMT > 4 mm strategy (reducing unnecessary biopsies) and than the EMT > 8 mm strategy (detecting more cancers). It identifies high-risk individuals in the EMT 4–8 mm group who would be missed by the EMT > 8 mm rule, without substantially expanding the biopsy pool. This strategy is particularly suitable for tertiary hospitals or specialized gynecology centers, where comprehensive risk assessment can be readily implemented, including platelet count measurement, diabetes status confirmation, and ensured access to follow-up care. By applying the ≥10% risk threshold strategy, only 4% of patients in our cohort would have required endometrial sampling. This approach maintains acceptable cancer detection rates while significantly reducing the biopsy burden, making it particularly suitable for resource-limited settings such as community clinics or primary care facilities.

This study has several limitations. First, its retrospective design and restriction to hospitalized patients may have introduced selection bias, potentially distorting estimates of EAH or EC prevalence in the full cohort and in subgroups. Second, the cohort’s mean BMI was 23.4 kg/m^2^, indicating a predominantly non-obese population; consequently, the results may not apply to severely obese individuals (BMI ≥ 30 kg/m^2^) and need validation in higher-BMI cohorts. Third, the findings are preliminary, and the predictive model requires internal and external validation before it can be considered for clinical application. Future work should recruit larger, prospective cohorts and include rigorous validation to clarify risk factors and incidence of EAH or EC in asymptomatic postmenopausal women.

## 5 Conclusion

Using multivariable Poisson regression with robust variance, we derived a preliminary risk model that incorporates EMT, diabetes, age at menopause and platelet count to estimate individual probabilities of EAH or EC in asymptomatic postmenopausal women. This exploratory tool may assist clinicians in more selectively considering endometrial biopsy, potentially reducing unnecessary procedures, but requires prospective validation before routine use.

## Data Availability

The raw data supporting the conclusions of this article will be made available by the authors, without undue reservation.
