# Supplementary material for: A risk-stratified model for predicting endometrial atypical hyperplasia and cancer to guide biopsy decisions in asymptomatic postmenopausal women
Source: Front Med (Lausanne). 2025 Dec 5;12:1707883. doi: 10.3389/fmed.2025.1707883 (PMC12714613; doi:10.3389/fmed.2025.1707883)
Supplement: Supplementary file 1 [file Supplementary_file_1.docx]

**Supplemental Table 1: Univariate Analysis of Association Between Hematological Parameters and Endometrial Atypical Hyperplasia and Cancer (EAH or EC) Risk**

|  | **Univariate analysis** | |
| --- | --- | --- |
| **Independent variable** | **RR [95%CI]** | **p** |
| **Coagulation-related** |  |  |
| Prothrombin Time - seconds | 0.95[0.51,1.77] | 0.866 |
| Thrombin Time - seconds | 0.92[0.59,1.43] | 0.717 |
| Thrombin Time Ratio | 0.12[0,44.83] | 0.485 |
| Activated Partial Thromboplastin Time - seconds | 1.06[0.98,1.15] | 0.173 |
| Activated Partial Thromboplastin Time Ratio | 4.07[0.37,44.88] | 0.252 |
| Fibrinogen - g/L | 1.46[0.94,2.26] | 0.089 |
| **Complete Blood Count-related** |  |  |
| **Leukocyte Series** |  |  |
| Neutrophil Percentage | 0.33[0,39.33] | 0.647 |
| Neutrophil Absolute Count (×10⁹/L) | 1.02[0.77,1.36] | 0.872 |
| Monocyte Percentage | 0.99[0.16,6.14] | 0.991 |
| Monocyte Absolute Count (×10⁹/L) | 8.86[0.91,86.56] | 0.061 |
| Basophil Percentage | 0[0,7.21e+34] | 0.223 |
| Basophil Absolute Count (×10⁹/L) | 0[0,23919263.65] | 0.351 |
| Eosinophil Percentage | 0.51[0,5010054.13] | 0.934 |
| Eosinophil Absolute Count (×10⁹/L) | 2.22[0.08,60.12] | 0.636 |
| White Blood Cell Count (×10⁹/L) | 1.05[0.84,1.31] | 0.686 |
| Immature Granulocyte Percentage | 2.7e+8.19[0,6.0e+30] | 0.459 |
| Immature Granulocyte Absolute Count (×10⁹/L) | 85.81[0.06,1.3e+5.66] | 0.233 |
| Lymphocyte Percentage | 0.87[0.1,7.39] | 0.896 |
| Lymphocyte Absolute Count (×10⁹/L) | 1.06[0.63,1.81] | 0.817 |
| Nucleated Red Blood Cell Count (×10⁹/L) | 0[0,1.39e+47] | 0.795 |
| **Erythrocyte Series** |  |  |
| Red Blood Cell Count (×10^12^/L) | 1.29[0.54,3.1] | 0.564 |
| Mean Corpuscular Volume | 0.96[0.92,1] | 0.056 |
| Red Cell Distribution Width - Coefficient of Variation | 0.93[0.27,3.23] | 0.913 |
| Hematocrit | 0[0,1.85] | 0.063 |
| Hemoglobin Concentration - g/L | 0.97[0.94,1] | 0.089 |
| Mean Corpuscular Hemoglobin Concentration - g/L | 0.99[0.95,1.04] | 0.807 |
| **Platelet Series** |  |  |
| Platelet Distribution Width - fL | 0.76[0.55,1.06] | 0.106 |
| Large Platelet Cell Ratio | 0.02[0,11.44] | 0.223 |
| Mean Platelet Volume - fL | 0.74[0.48,1.14] | 0.169 |
| Plateletcrit | 232.76[0.16,3e+5.3] | 0.142 |

**Supplemental Table 2: Adjusted Relative Risk of Endometrial Atypical Hyperplasia or Cancer (EAH or EC) for Endometrial Thickness >8 mm Across Different Statistical Models**

| **Adjustment Model** | **aRR (95% CI) for EMT >8mm** |
| --- | --- |
| Adjusted demographic factors* | 18.81[5.447,64.924] |
| Adjusted metabolic & hormonal factors† | 13.07[4.455,38.365] |
| Adjusted full factors‡ | 7.90[2.255,27.644] |
| Main analysis model§ | 12.27[4.24,35.45] |

aRR, adjusted relative risk; CI, confidence interval; EMT, endometrial thickness; BMI, body mass index.

* Age, Ethnicity, Occupation, Education level.

† Age at menopause, Time since menopause, BMI, Diabetes, Hypertension, Hormone replacement therapy.

‡ All variables considered.

§ Includes all statistically significant variables from univariate analysis (EMT, diabetes, age at menopause, platelet count).

**Supplemental Table 3: Diagnostic Performance of Different Endometrial Biopsy Strategies for Detecting Endometrial Atypical Hyperplasia or Cancer in Asymptomatic Postmenopausal Women (N=928)**

| **Biopsy Strategy** | **Total Biopsies Performed (n)** | **Positive Cases Detected (n)** | **Sensitivity** | **Specificity** | **PPV** | **NPV** |
| --- | --- | --- | --- | --- | --- | --- |
| **EMT >4 mm strategy** | 343 | 19 | 79.2% | 64.2% | 5.54% | 99.15% |
| **EMT >8 mm strategy** | 153 | 16 | 66.7% | 84.8% | 10.46% | 98.97% |
| **>5% risk-stratified model** | 158 | 17 | 70.8% | 84.4% | 10.76% | 99.09% |
| **>10% risk-stratified model** | 40 | 11 | 45.8% | 96.8% | 27.50% | 98.54% |

EMT, endometrial thickness; PPV, positive predictive value; NPV, negative predictive value.

**Supplemental Table 4: Variance Inflation Factors (VIF) for the Analyzed Variables**

|  | **Tolerance** | **VIF** |
| --- | --- | --- |
| **EMT** | 1.000 | 1.000 |
| **Diabetes** | 0.998 | 1.002 |
| **Age at menopause(years)** | 0.997 | 1.003 |
| **Platelet count(10⁹/L)** | 0.998 | 1.002 |

Abbreviations: EMT, endometrial thickness; VIF, variance inflation factors.
